# Development of ZnCdSe/ZnS quantum dot-based fluorescence immunochromatographic assay for the rapid visual and quantitative detection 25⁃hydroxyvitamins D in human serum

**DOI:** 10.3389/fbioe.2023.1326254

**Published:** 2023-12-22

**Authors:** Jianfa Wang, Guoshao Sun, Fang Li, Zhi Zhu, Lei Sun, Pengju Lv, Han Yue

**Affiliations:** ^1^ Department of Orthopedics, Zhengzhou Central Hospital Affiliated Zhengzhou University, Zhengzhou, Henan, China; ^2^ Center of Stem Cell and Regenerative Medicine, Zhengzhou Central Hospital Affiliated Zhengzhou University, Zhengzhou, Henan, China

**Keywords:** 25-hydroxyvitamin D, ZnCdSe/ZnS, quantum dots, fluorescence immunochromatographic assay, quantitative detection

## Abstract

Vitamin D deficiency is associated with various diseases such as obesity, digestive problems, osteoporosis, depression, and infections, and has therefore emerged as a topic of great interest in public healthcare. The quantitative assessment of 25-hydroxyvitamin D (25-OH VD) in human serum may accurately reflect the nutritional status of vitamin D in the human body, which is significant for the prevention and treatment of vitamin D-deficient patients. In this study, we developed an assay for quantitative detection of 25-OH VD based on the 25-OH VD monoclonal antibody (mAb), and identified the optimal process parameters. The following process settings were found to be suitable for the test strips: pH of 7.6, 1-Ethyl-3-(3-dimethylaminopropyl) carbodiimide (EDC) ratio of 1:2000, and the anti-25-OH VD mAb ratio was 1:8. The equilibration time of the immune dynamic assay was 15 min. Under optimal conditions, the quantum dot nanoparticle-based fluorescent immunochromatographic assay (QDs-FICA) exhibited dynamic linear detection of 25-OH VD in PBS, from 5 ng/mL to 100 ng/mL, and the strip quantitative curve could be represented by the following regression equation: y = −0.02088 logx)+1.444 (R2 = 0.9050). The IC50 of the QDs-FICA was 39.6 ± 1.33 ng/mL. The specificity of the QDs-FICA was evaluated by running several structurally related analogues, including 25-OH VD_2_, 25-OH VD_3_, 1,25-OH_2_VD_3_, 1,25-OH_2_VD_2_, VD_2_, and VD_3_. The coefficients of variation were all below 10%. The shelf life of the test strips in this study was about 160 days at room temperature. Briefly, this study is the first to perform QDs-FICA for the rapid visual and quantitative detection of 25-OH VD, with great potential significance for clinical diagnosis of vitamin D-associated diseases.

## Introduction

Vitamin D is an essential fat-soluble steroid derivative that exerts its physiological effects after undergoing hydroxylation twice and binding specifically to the intracellular vitamin D receptor (VDR) (Janjusevic et al., 2021). 25-Hydroxyvitamin D was first reported as a marker of bone metabolism in a Nordic study (Revez et al., 2020). Studies have found that a lack of adequate sunlight exposure can cause serious disruption to the normal growth of adolescents and even lead to rickets (McIntosh et al., 1982; Bouillon et al., 2022). Furthermore, in middle-aged and elderly people, vitamin D deficiency can result in osteoporosis, which can easily lead to fractures and other problems (Reid et al., 2014). In addition, studies have shown that vitamin D deficiency can cause diseases related to the skeletal system, cardiovascular system, glucose metabolism, nervous system, muscular system, cell proliferation, immune system, endocrine system, and tumorigenesis (Di Somma et al., 2017; Liu et al., 2018; Gouni-Berthold and Berthold, 2021).

The biomarkers of bone metabolism, namely, osteocalcin (BGP), parathyroid hormone (PTH), 25-OH VD, and type 1 procollagen amino terminal extender peptide (P1NP) are sensitive, specific, and non-invasive clinical aids to the diagnosis of osteoporosis, and can enable accurate evaluation of bone turnover at the level of intact bone tissue (Balsan et al., 1986). Among them, 25-OH VD is now widely used in the experimental diagnosis of osteoporosis and the assessment of growth and development of children (Garabédian et al., 1983; Holick, 2023). For vitamin D-deprived individuals who take high-dose vitamin D supplementation, it is even more important to regularly and accurately monitor 25-OH VD levels in the body to prevent overdose and consequent toxicity (Vieth, 1999; Binkley et al., 2010). Serum 25-OH VD concentration testing has been found to be the most reasonable and reliable indicator of overall vitamin D status (Mano et al., 2023). The deficiency of 25-OH VD is highly prevalent worldwide; as a result, regular testing of 25-OH VD levels to ensure its adequacy is important for disease prevention.

The current 25-OH VD assay methodology is based on liquid chromatography-tandem mass spectrometry (LC-MS/MS) and ELISA (Su et al., 2014; Yun et al., 2015). LC-MS/MS is recognized as the gold standard for assays and is also used as a reference for validation of other assays, with very high sensitivity, specificity, and accuracy. However, the instruments used are expensive, require high professionalism of operators, and require self-developed assays. At present, most hospitals in China are not equipped with the required instruments and professional staff, which limits the clinical application of LC-MS/MS methods. Fluorescence immunochromatographic assay (FICA) is a rapid diagnostic technique developed in the early 1980s, which is a classical rapid test based on the immunoreaction of antigens and antibodies (Lin et al., 2015). Simultaneously, Compared with LC⁃MS/MS methods, immunochromatography is easy and fast to operate. Quantum dots (QDs) have attracted wide interest in bioimaging and biosensing (Geszke-Moritz and Moritz, 2013; Yang et al., 2022). Because of their unique optoelectronic properties relative to traditional labeling reagents (organic and protein-based fluorophores), and significant progress has been made in the development of complex surface-coating technologies (Monteiro et al., 2017; Koo et al., 2022). Applications in clinical diagnostics, food safety, environmental protection, and pesticide residues are widespread.

Quantum dot rapid immunochromatographic assay is a new labeled immunoassay technique combining luminescence reaction with immunoassay for the detection of trace antigens or antibodies. This technique has the advantages of high stability, good reproducibility, high sensitivity, high specificity, and rapid detection (Wang et al., 2021; Chen et al., 2022; Su et al., 2022). T The immunoassay is suitable to be carried out in mid-level and primary hospitals because of the small amount of sample used, relatively inexpensive assay equipment, and simple instrument operation (Li et al., 2022). In the rapid development of point-of-care tests (POCT), user needs such as test accuracy, sensitivity, and stability are high. In this context, fluorescence immunochromatography technology meets the need for rapid detection and is easy to operate (Ahmad Najib et al., 2022; Singh et al., 2020; Zhang et al., 2019). Therefore, in this study, a quantum dot nanoparticle-based fluorescent immunochromatographic assay (QDs-FICA) for the detection of 25-OH VD was established using the quantum dot rapid immunochromatographic technique. The assay was tested and found to be extremely sensitive and specific, with potential utility in the serological detection of 25-OH VD and clinical application for the diagnosis of vitamin D-related diseases.

## Materials and methods

### Materials and clinical samples

Carboxy water-soluble QDs-COOH (ZnCdSe/ZnS, core/shell) with a size of 12 nm were obtained from Wuhan Jiayuan Quantum Dots Corporation, Ltd. (Wuhan, China). Staphylococcal protein-A (SPA) was purchased from Beijing Solarbio Science & Technology Corporation, Ltd. Fetal calf serum (FCS) was bought from Tian hang Biological Technology Co., Ltd. (Zhejiang, China). The following substances were purchased from Sigma-Aldrich Chemical Co. (St. Louis, MO): 25-OH VD antigen, bovine serum albumin (BSA), staphylococcal protein-A (SPA), and N-(3-dimethylaminopropyl)-Nʹ-ethylcarbodiimide hydrochloride (EDC). 25-OH VD monoclonal antibody was purchased from Bioventix Biotechnology, United Kingdom. Vitamin D and 25-OH VD standards were purchased from Hanzun Biological Co., LTD. 25-OH VD derivative were purchased from Xiamen Tongrenxin Gong Department. The nitrocellulose membrane, sample pad, backing card, absorbent pad and the filter membrane were from Millipore, US. The commercial Chemiluminescence Immunoassay (CLIA) kit for 25-OH VD quantitative analysis was from Immunodiagnostic Systems Holdings PLC (IDS), United Kingdom. Millipore’s Milli-Q filtration system was used to create ultrapure water. One-hundred clinical samples were collected from patients who visited the outpatient clinic or physical examination center of Zhengzhou Central Hospital in 2023. Each patient underwent a health interview and was asked to sign an informed consent form.

### Bioconjugation QDs-COOH with 25-OH VD mAb

The synthesis of QDs-mAb conjugates was carried out as described in an earlier study (Zhou et al., 2021). In this study, improved synthesis conditions were used for the 25-OH VD probes. A reaction vessel was filled with 12.5 μL of the QDs-COOH (ZnCdSe/ZnS) (8 μM) and 30 μL of the EDC (4.0 mg/mL), and the mixture was incubated for 30 min at room temperature 25°C. EDC solution was then used to activate the QDs-COOH. Next, 30 μL of 25-OH VD mAb (4.0 mg/mL) in borate-buffered saline (BBS, 10 mM, pH = 7.6) was added to the reaction. A shaking incubator was used to allow the mixture to continue to react for an additional 2 hours at 25°C. At the end of the reaction, the reaction was centrifuged at 8,000 rpm for 3 min to remove any agglomerates and supernatant was discarded. The sample was concentrated and purified five times using ultrafiltration tubes, and the final product was redissolved in the appropriate target coupling buffer. QDs-mAb conjugates were saved in a new brown microcentrifuge tube and stored at 4°C for future use.

### Preparation of the QDs-FICA strip

The QDs-FICA consists of five main parts: sample pad, conjugate pad, nitrocellulose membrane, absorbent pad, and backing card. The 25-OH VD-BSA conjugate and SPA solution were immobilized on NC membranes as the test T) and control C) lines using a BioDot XYZ3050 dispensing platform (Irvine, CA, United States). The NC membranes was then dried in an electric blast oven at 40°C for 4 hours. The distance between the T and C lines was roughly 5 mm. Next, the test strips were assembled in the order of the sample pad, conjugate pad, NC membrane, and absorption pad. They were then packaged in a plastic cassette after being sliced into 2.8 mm-wide test strips using a BioDot CM 4000 Guillotine Cutter. They were then stored with desiccant and sealed in aluminum foil at 4°C.

### Optimization of conjugating conditions

The inputs of EDC, 25-OH VD mAb, and pH value play a crucial role during the conjugation process. These conditions were optimized by an orthogonal experiment, which is described in Table 1. The fluorescence intensity on the test paper and the peaks of the fluorescence spectral curves were recorded using an MD-980 Multi-channel Fluorescent Immunoassay Analyzer (Micro detection Corporation, Ltd., Nanjing, China). The measurements were used to determine the best set of conjugation conditions.

**TABLE 1:** Optimization of the concentration of 25-OH VD-BSA and the d the volume of QB-mAbs by using a checkerboard titration (n = 3).

NO	Concentration of 25-OH VD-BSA (mg/mL)	Volume of QD-mAbs	FI_T_	FT_C_	FI_T_/FI_C_	Inhibition ratio (%)
1	0.2	1.0	1,260 ± 62	1,556 ± 268	0.82 ± 0.10	72.88 ± 0.03
2	0.2	1.5	1,469 ± 70	1,507 ± 89	1.01 ± 0.06	60.97 ± 0.05
3	0.2	2.0	2,306 ± 39	1,318 ± 41	1.74 ± 0.04	71.24 ± 0.01
4^a^	0.4	1.0	642 ± 23	815 ± 12	0.78 ± 0.03	80.27 ± 0.02
5	0.4	1.5	918 ± 40	1,168 ± 58	0.78 ± 0.02	72.94 ± 0.02
6	0.4	2.0	1,123 ± 115	1,108 ± 42	1.12 ± 0.04	63.39 ± 0.04
7	0.8	1.0	765 ± 28	681 ± 19	0.97 ± 0.01	38.05 ± 0.07
8	0.8	1.5	1725 ± 87	987 ± 88	1.75 ± 0.12	72.78 ± 0.01
9	0.8	2.0	1,151 ± 79	645 ± 41	1.78 ± 0.16	74.32 ± 0.03

^a^ The optimal condition under the concentration of 1.0 ng/mL serum 25-OH VD.

### Standardizing 25-OH VD QDs-FICA

The 25-OH VD stock solution was diluted with 0.01 mol/L PBS buffer solution to achieve final concentrations of 0, 5, 10, 20, 30, 40, 60, 80, and 100 ng/mL. Then, 100 μL of each standard solution was incubated for 3 min with 3 μL of standard immunoprobe before being applied to the spiking wells of the test strips. The T/C ratios of the strips were measured using a gold standard reader after 15 min. Each concentration was tested five times. The sensitivity, IC_50_ value, and linear quantification range of 25-OH VD immunochromatographic strips were determined by plotting the standard curve with the logarithm of 25-OH VD concentration as the horizontal coordinate and the T/C value as the vertical coordinate. The sensitivity was computed by subtracting the mean value for the negative samples from their triple standard deviation.

### Limit of detection the QDs-FICA

The 25-OH VD stock solution was diluted with 0.01 mol/L PBS buffer solution to the series standard concentration of 0, 12.5, 5, 7.5, 15, 25, 50, 60, 80, 100, 125, 130, 140, and 150 ng/mL. The FI_T_/FI_C_ ratio of the test strip was detected using the fluorescence instrument, and each concentration was measured in parallel five times. The FI_T_/FI_C_ ratio of the test strip with a concentration of 0 ng/mL of added 25-OH VD was denoted as B0 and the FIT/FIc ratios of the other added concentration was denoted as B. The competition inhibition curve of 25-OH VD strips with PBS as the sample matrix was plotted by logarithmic mapping of the B/B0 value of the 25-OH VD concentration, and the IC_50_ value and linear quantitative range of the strips were determined at this time.

### Cross-reactivity of the QDs-FICA

To evaluate the specificity of 25-OH VD colloidal gold quantitative test strips, six common 25-OH VD structural analogs, namely, 25-OH VD_2_, 25-OH VD_3,_ 125-OH_2_VD_3_, 125-OH_2_VD_2_, VD2, and VD3, were selected for cross-reaction experiments. A series of standards concentration with final concentrations of 0, 5, 10, 20, 30, 40, 60, 80, and 100 ng/L were prepared in the stock solution, and the above solutions were detected with 25-OH VD colloidal gold strips. Each concentration was measured three times in parallel, and the standard curve was drawn according to the T/C ratio measured by the colloidal gold reader. The IC_50_ of each competitor was obtained, and the cross-reaction rate of each analogue with 25-OH VD was calculated according to the following formula: (Cr) %= (25-OH VD IC_50_) (cross analogue IC_50_) 100%.

### Interruptibility of the QDs-FICA

The test was repeated twice with the same dilution of the interference test samples; then, the samples of low, medium, and high concentration levels were used as the base samples. We divided each base sample into five portions, one of which was added to the sample dilution, without interfering substances, as the control sample. The other four portions were added in equal volumes, with different concentrations of interfering substances, as the analysis sample. The measurement was repeated three times to determine the average value of each sample to calculate the interference rate, using the following formula: Interference rate = (average concentration of analyzed sample average concentration of base sample)/average concentration of base sample × 100%. The interference effect between the addition of interferon and the absence of interferon was calculated, and ≤10% bias was used as the judgment standard.

### Accuracy and precision of the QDs-FICA

The 25-OH VD standard was added into 0.01 mol/mL PBS buffer solution until the final concentration was 25, 50, and 100 ng/mL. The same batch of test strips was used to detect the three concentrations of low, medium, and high levels; measurement was performed every 3 days and repeated three times for each sample. To evaluate the accuracy and precision of the test strips, the recovery rate of each concentration and the difference between batches of the test strips was calculated.

### Stability of the QDs-FICA

Stability experiments were performed by randomly selecting 100 test strips from the same batch and placing them in the oven at 60.1°C. The experiments were designed according to the Arrhenius formula, the time intervals for which 60.1°C needs to be tested are the Day 0, Day 1, Day 2, Day 4 and Day 8 (about 0 days, 40 days, 80 days, 160 days, and 320 days at 25°C). Three standard antibody dilutions of 1:500, 1:2,000, and 1:5,000 were measured and three parallels were set up for each sample. The average FI_T_/FI_C_ values were calculated. The QDs solution with a concentration of 0.5 mg/mL was configured and directly scribed to the T-line position of the NC membrane using a membrane scribe, assembled into a test card and then placed in the tester for 100 consecutive readings to compare the average lifetimes of the QDs and mAb QDs.

### QDs-FICA for 25-OH VD qualitative and quantitative detection

For construction of the quantitative standard curve for 25-OH VD detection, the extract was mixed with 25-OH VD to make standard solutions (0, 0.01, 0.05, 0.1, 0.5, 1.0, 2.5, 5.0, 10, and 50 ng/mL). 25-OH VD standard solution detection was performed using fluorescent test strips. The cut-off value, i.e., the lowest 25-OH VD concentration that caused T line fluorescent band invisibility, was used to evaluate the qualitative performance of QDs-FICA. The T line fluorescence intensity dropped as the 25-OH VD concentration increased, disappearing under a handheld UV light. The MD-800 multiple immunochromatographic test strip analyzer measured the T line, C line, and FI ratio (FI_T_/FI_C_) fluorescence intensity three times for each standard test for quantitative detection.

## Results

### Characterization of QDs and mAb-QDs conjugates

**FIGURE 1:**
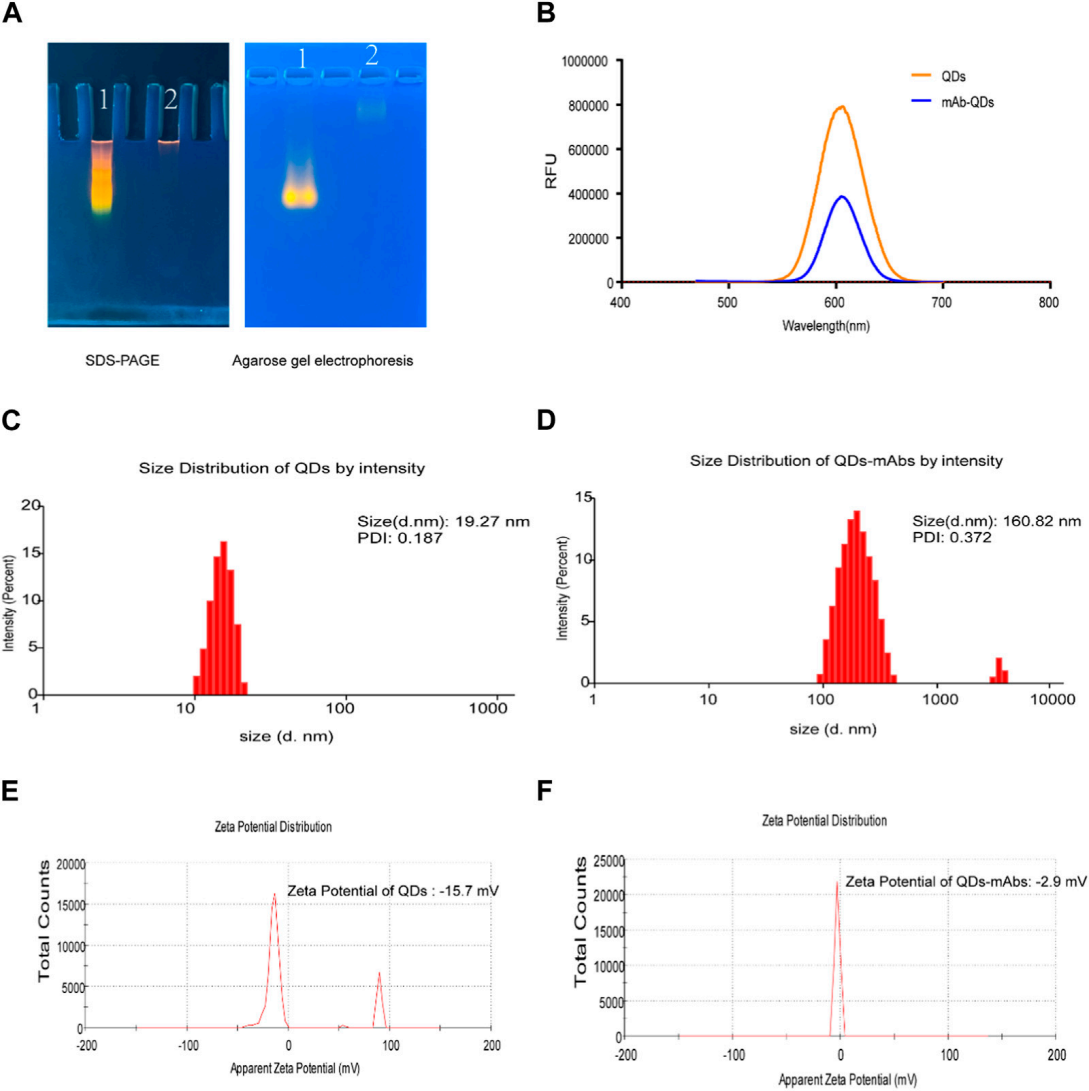
Fluorescent Probe Validation. **(A)** SDS-PAGE and Agarose gel electrophoresis. 1. QDs; 2. Anti-25-OH VD-mAb-QDs. **(B)** QDs and anti-25-OH VD-mAb-QDs fluorescence emission spectrum (Excitation wavelength 450nm, emission wavelength 585 nm). **(C, D)** Hydrodynamic diameter of the QDs and mAb-QDs by a Malvern laser particle size analyzer. **(E, F)** Size and ζ potential distributions of the QDs and anti-25-OH VD-mAb-QDs by a Malvern laser particle size analyzer.

The QDs were synthesized by encapsulating ZnCdSe/ZnS using a microemulsion method as previously reported (Shen et al., 2015). Agarose gel electrophoresis (AGE) and sodium dodecyl sulfate polyacrylamide gel electrophoresis (SDS-PAGE) experiments indicated that anti-25-OH VD-mAb-QDs have a large molecular weight, which slows down their migration in agarose gels (Figure 1A). When anti-25-OH VD-mAb were coupled to QDs, the excitation and emission wavelengths remained unchanged at about 605 nm, but the fluorescence intensity was weaker than that of QDs, which may be attributed to the effect of the antibody on fluorescence detection or the occurrence of fluorescence burst during the coupling process (Figure 1B). The particle size distribution and potential variation of the nanoparticles were measured by dynamic light scattering (DLS), as shown in (Figures 1C,D), The mAb-QDs conjugates reached a size of 160.82 nm, and the polymer dispersity index (PDI) value of mAb-QDs conjugates was 0.372. In comparison, the QDs were only 19.27 nm in size, and the PDI value of the QDs was 0.187. As a result, the size of the hydrated QDs was significantly smaller than that of the mAb-QDs. Furthermore, Figures 1E,F show the zeta potential (ζ potential) values of the anti-25-OH VD-mAb-QDs conjugates and QDs. While the potential of QDs was detected by DLS to be −15.7mV, the potential of anti-25-OH VD-mAb-QDs was −2.9 mV. Transmission electron microscopy (TEM) images showed (Supplementary Figure S1) that the prepared anti-25-OH VD-mAb-QDs had a regular spherical shape with a relatively uniform particle size distribution. High-resolution TEM images of individual particles showed that a large number of oil-soluble ZnCdSe/ZnS QDs were tightly embedded in the polymer. The above several sets of data indicate that there is effective coupling between the QDs and anti-25-OH VD-mAb, confirming successful synthesis of the immunofluorescence probe.

### Detection of 25-OH VD using QDs-FICA platform

**FIGURE 2:**
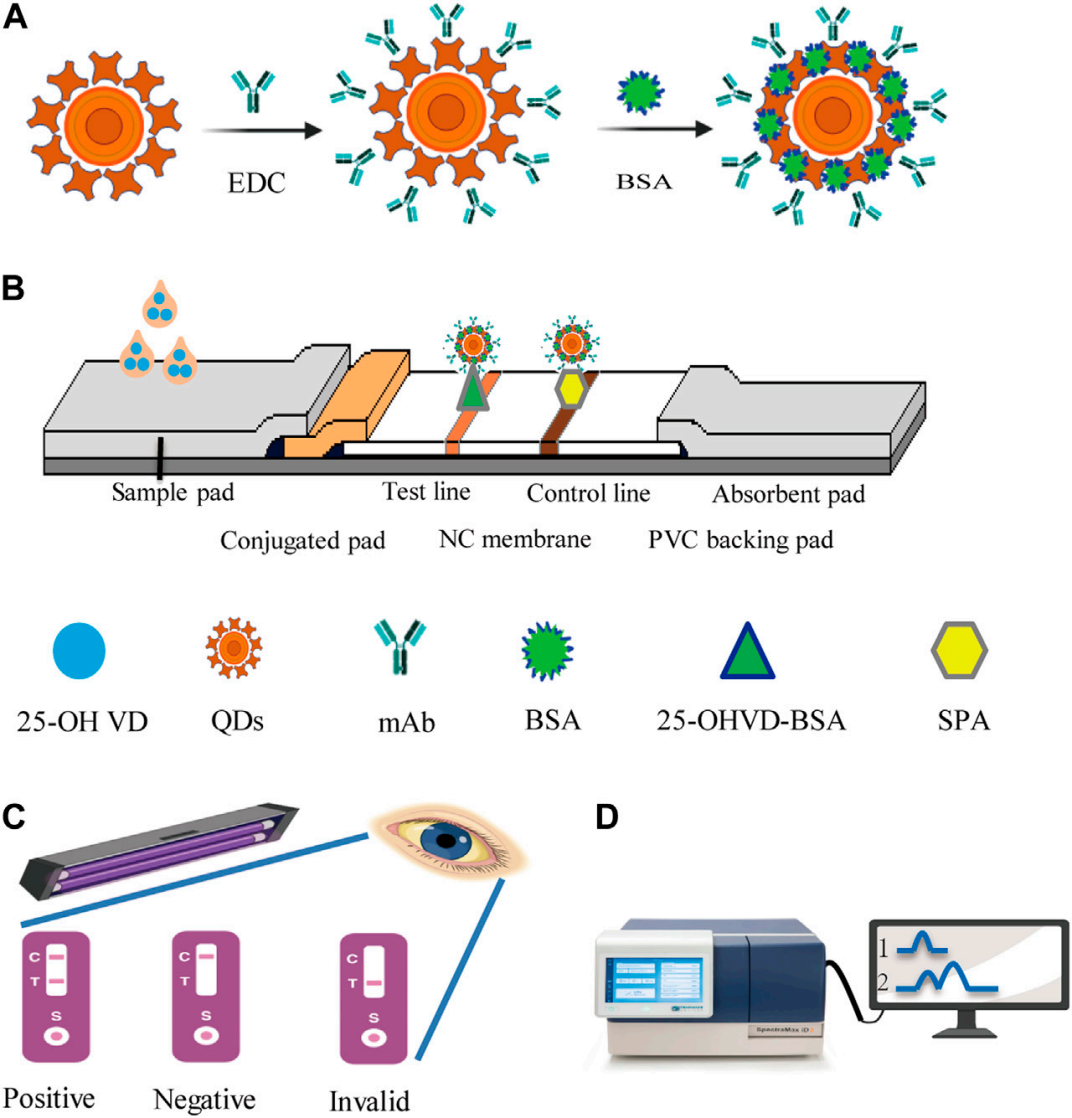
Schematic representation of the sandwich procedure for the detection of 25-OH VD using QDs-ICA platform. **(A)** Schematic illustration of the anti-25-OH VD-mAb-QDs probes preparation. **(B)** The five components assembly of conventional ICA sensor, the positive tests consequence consisting of a test line (T) and a control line (C). **(C)** Immunochromatographic assay shows a negative result with the presence of control line. **(D)** The fluorescence strip readera. 1, QDs; 2 anti-25-OH VD-mAb-QDs.

QDs-FICA is a straightforward and visually appealing approach for validating the bioactivity of mAb-QDs. A schematic illustration for the detection of 25-OH VD using the QDs-FICA-based platform is shown in Figure 2. Briefly, QDs were used to replace colloidal gold particles as signal markers. QDs were coupled with the corresponding antibody of 25-OH VD to be tested, and then sprayed on the binding pad. Immunochromatographic test strips were made by assembling the sample pad, the binding pad, the reaction membrane, and the absorbent pad. The FICA technique revealed that QDs could not selectively interact with the T line (25-OH VD-BSA) and C line (SPA). However, anti-25-OH VD-mAb -QDs underwent strong interactions with the C and T lines when exposed to ultraviolet light from a portable UV lamp with an excitation wavelength of 365 nm.

### Optimization of coupling conditions

**FIGURE 3:**
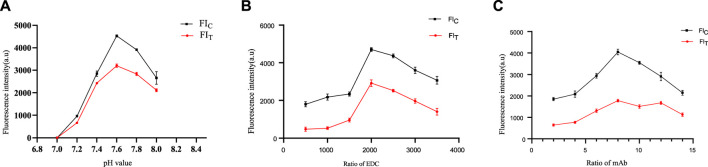
Optimization of coupling conditions for QDs-mAb. **(A)** Determine the optimal pH of the label. **(B)** Determine the optimal amount of label led activator EDC. **(C)** Determine the optimal amount of labelled anti-25-OH VD-mAb.

Because of the pH, EDC and anti-25-OH VD mAbs can have a significant effect on the stability and biological activity of the conjugate. Therefore, coupling conditions were optimized in this study. As shown in Figure 3A, when the pH was 7.6, the highest values of both FI_T_ and FI_C_ were obtained. When the molar ratio of QDs to EDC is 1:2000, FI_T_ and FI_C_ are saturated (Figure 3B). The excessive amount of EDC would lead to a large amount of conjugate aggregation, which is unfavorable for mutual coupling. In addition, the results showed that FI_T_ and FI_C_ were saturated when the molar ratio of QDs to the anti-25-OH VD mAb was 1:8 (Figure 3C). The optimal coupling conditions were as follows: a pH of 7.6, an EDC ratio of 1:2000, and a 25-OH VD:mAb ratio was 1:8.

### Optimization of the QDs-FICA

The dosages of 25-OH VD-BSA and anti-25-OH VD mAb-QDs are particularly important for optimal sensitivity and fluorescence intensity of the FICA. The experimental results are presented in Table 1. Which shows that the fluorescence intensity of the T and C lines of the test strips was stronger when the concentration of 25-OH VD-BSA was 0.4 mg/mL and the immunoprobe dosage was 1.0 μL (group 4), The FI_T_ and FI_C_ were 642 ± 23 and 815 ± 12, respectively. The competition inhibition rate of 1.0 ng/mL positive samples was as high as 80.27 ± 0.02 (n = 3), which indicated the best detection effect relative to the rest of the groups. Therefore, the optimization results for group 4 were determined as the best quantitative analysis conditions for the test strips.

### Optimization of detection conditions

**FIGURE 4:**
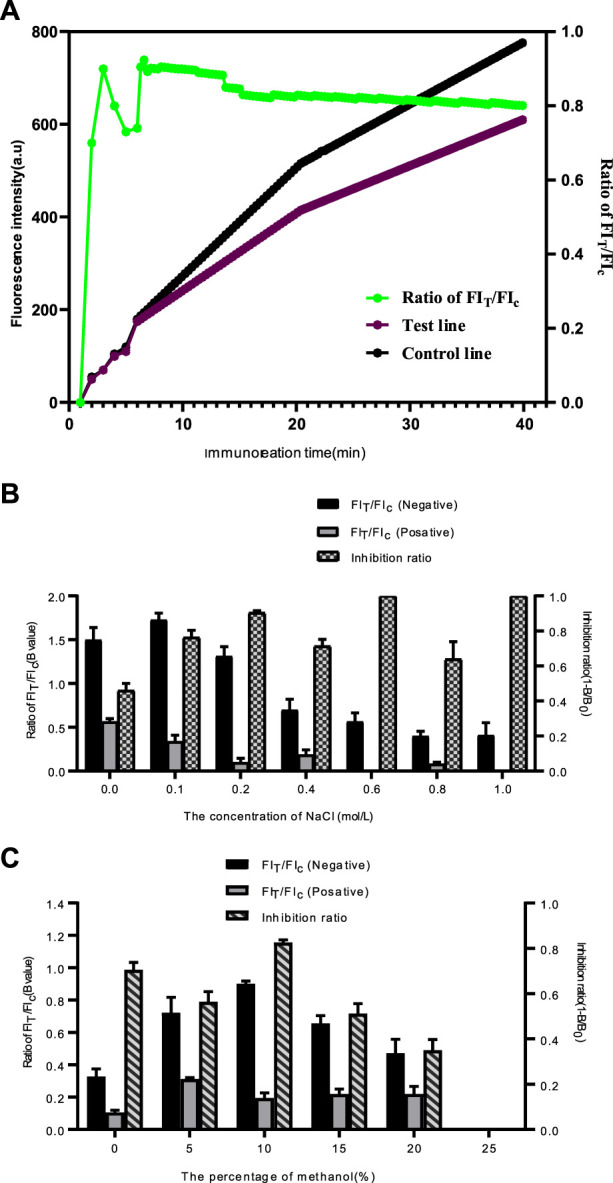
Optimization detection conditions of the QDs-FICA. **(A)** Kinetic curves of immunoreaction on test line, control line and FI_T_/FI_C_ of QDs-based immunochromatographic strip. **(B)** Effect of ion strength in samples on the T/C ratio. **(C)** Effect of ethanol in samples on FI_T_/FI_C_ and competitive inhibition rate.

In order to achieve the best performance of the QDs-FICA platform, we further optimised the immunoreaction time of the assay, the salt ions and the ethanol content of the sample solution. As shown in Figure 4A, The FI_T_ and FI_C_ improved continuously within 45 min The fluorescence signals of T and C lines were gradually strengthened after 10 min of spiking, and the FI_T_/FIc ratio tended to be 0.8 when the immunoreaction time reached 15 min. Specific experimental results are shown in Figure 4B. When the salt ion concentration is 0.2 mol/L, the T/C value of the negative sample is 1.365, and the T/C value of the positive sample is 0.028. The competitive inhibition rate reaches 92.5%. The maximum values of FI_T_/FI_C_ for negative samples and competitive inhibition for positive samples were reached when the concentration of ethanol in the samples was 10%. When the concentration of ethanol is greater than 10%, FI_T_/FI_C_ and the competitive inhibition rate decrease rapidly (Figure 4C). The reason is that the concentration of organic solution exceeds the capacity of protein, which will lead to the loss of protein activity.

### Sensitivity and stability of the QDs-FICA

The stability results showed that the detection efficiency of the test strips stored at 60.1°C for 4 days (160 days, 25°C) was weakened, and only 1:500 high concentration antibody standards could be measured. So it was inferred that the storage time of the test strips in this study was about 160 days at room temperature. See Supplementary Figure S2. The fluorescence lifetime measurement results show that although the fluorescence intensity of anti-25-OH VD-mAb-QDs is slightly lower than that of QDs, the fluorescence intensity of 100 consecutive measurements of the T-line remains basically unchanged. This demonstrates that the lifetime of anti-25-OH VD-mAb-QDs can satisfy normal clinical detection applications (Supplementary Figure S3).

### Analytical performance and validation of QDs-FICA

**FIGURE 5:**
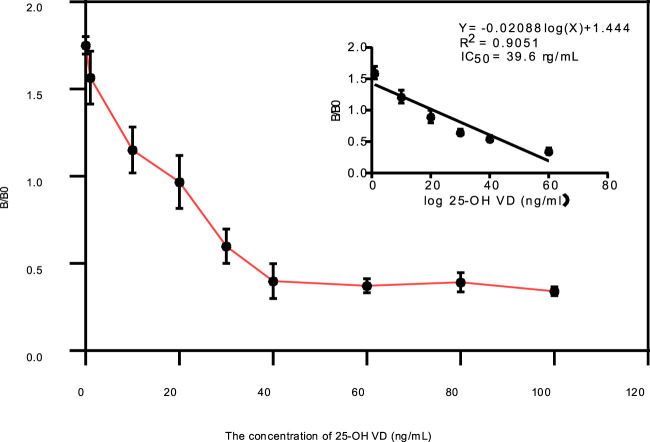
Evaluation of the test strips using 25-OH VD standards, Calibration curve for quantitative detection of 25-OH VD by QDs-FICA.

The standard curve was shown in Figure 5, its linear regression equation is y = −0.02088 logx)+1.444 (*R* ^2^ = 0.9050). The concentration of 25-OH VD shows a very good linear relationship between 5 ng/mL and 100 g/mL, according to the linear regression equation. It is concluded that the IC_50_ of the test strip was 39.6 ± 1.33 ng/mL (n = 3). Table 2 showed the cross-reaction rates of 25-OH VD_3,_ 125-OH_2_VD_3_, and 125-OH_2_VD_2_ with the strip were 26%, 11%, and 25%, respectively. The cross-reaction rates of VD_3_ and VD_2_ were less than 1% with the test strip. Therefore, the fluorescence test strip has good specificity.

**TABLE 2:** Specificity tests against structurally related analogies.

Name	Cross-reaction rate (%)
25-OH VD2	100
25-OH VD3	26
125-OH2VD3	11
125-OH2VD2	25
VD2	<1
VD3	<1

As shown in Supplementary Table S1, the interference rate is less than 8%. This indicates that the method is more resistant to strong interference. The test results for the 25-OH VD immunochromatographic strips between batches and within batches are shown in Table 3. The recovery rates of 25-OH VD addition samples at different concentrations in the test strip range from 80.48% to 93.41%, and the coefficient of variation for the test strip ranged from 1.78% to 9.96%. The recoveries of the 25-OH VD addition samples were 85.35%–100.65%, and the coefficient of variation was between 2.39% and 9.35%. The results show that the 25-OH VD QD fluorescent microsphere strip has good precision and accuracy.

**TABLE 3:** Accuracy and precision of the QD immunochromatographic test strip.

Spiked 25-OHVD (ng/mL)	Intra-assay			Inter-assay^a^		
	Test^b^	CV (%)	Recovery (%)	Test^b^	CV (%)	Recovery (%)
20	20.12 ± 0.019	1.78	80.48	21.34 ± 0048	4.34	85.35
50	46.09 ± 0.014	1.84	92.18	50.13 ± 0.019	2.39	100.26
100	93.41 ± 0.057	9.96	93.41	100.65 ± 0.054	9.35	10,065

^a^ Assay was completed every 3 days for 15 days continuously.

^b^ Mean value of three replicates at each 25-OHVD, spiked concentration.

### Comparison of QDs-FICA and commercial chemiluminescence immunoassay (CLIA) methodology

**FIGURE 6:**
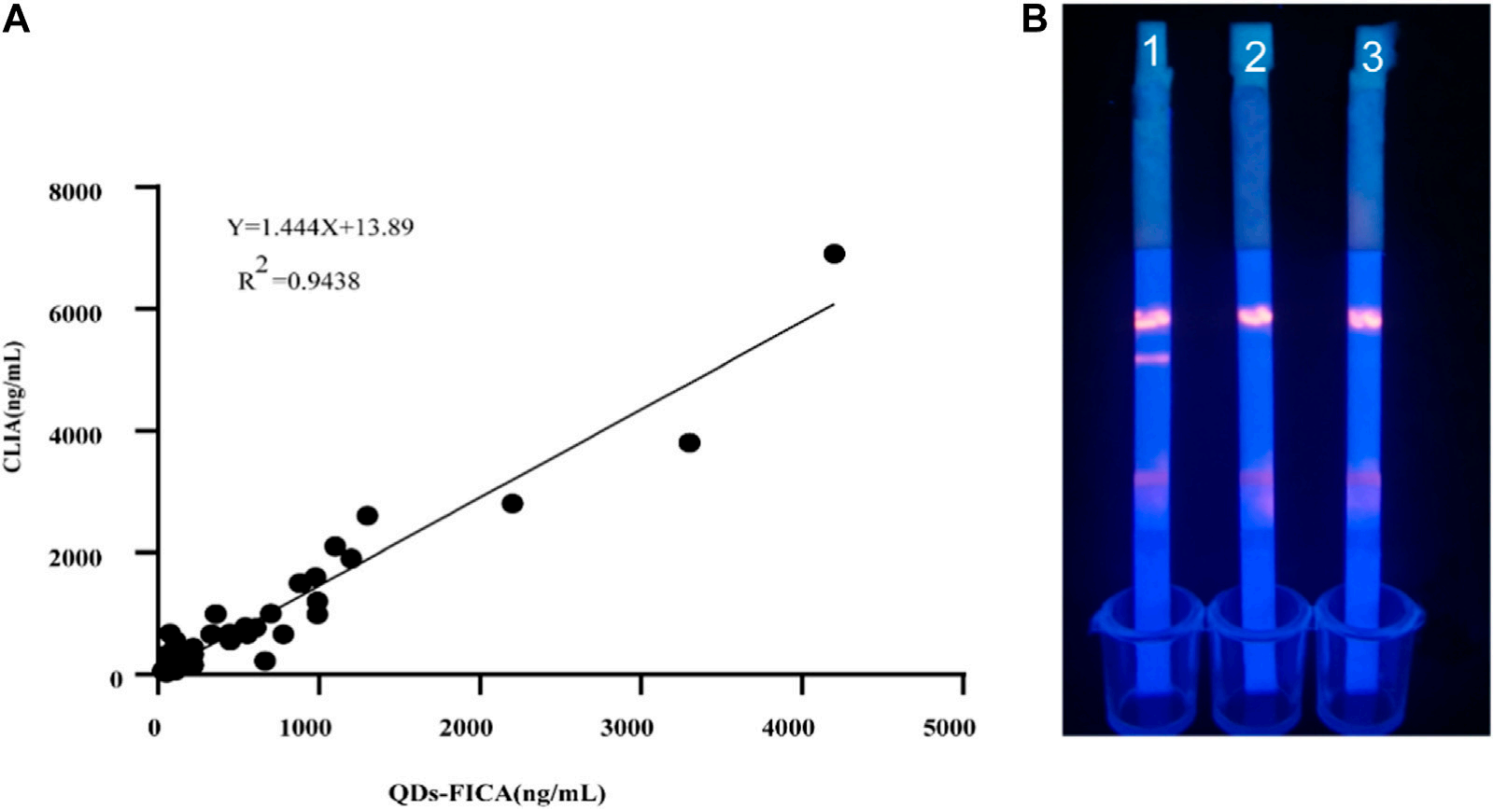
Evaluation of the QDs-FICA platform with clinical sample. **(A)** 25-OH V D-positive serum samples were quantified using both QDs-FICA and a commercially available human 25-OH VD CLIA kit. **(B)** QDs-FICA visual readout. 1, positive sample; 2, negative sample; CK, blank control with double-distilled water.

We collected 100 human serum samples from Zhengzhou Central Hospital. For comparison with the chemiluminescence immunoassay method, a semi-quantitative commercial ELISA kit was purchased from DiaSorin. The results were presented in Figures 6A, B, the serum results were compared with the results for the control reagents. The regression equation was Y = 1.455X+13.89, and the correlation with DiaSorin was *R* ^2^ = 0.9438. The positive samples containing a high viral load showed color very quickly (5–10 min) at the T lines; this was statistically significant, and confirms that the results of the tests are in good agreement.

## Discussion

Vitamin D has become more popular in recent years as people’s living circumstances have improved (Aloia et al., 2010). Vitamin D is one of the most vital nutrients for maintaining human life activities. UV radiation exposure converts 7-dehydrocholesterol in the skin to vitamin D, which then plays a crucial function in blood circulation throughout the body (Ahmed et al., 2020). At present, the methodology involved in the 25-OH VD detection kit mainly includes six methods (Tripathi et al., 2022). Because 25-OH VD is not found in free form in human serum, but rather in conjunction with vitamin D binding protein (VDBP), the test strips are utilized to evaluate genuine human blood samples (Zhu et al., 2022). LC-MS/MS method instruments are expensive and have strict installation conditions, requiring a larger area to meet the requirements (Ko et al., 2021; Gao et al., 2022). In recent years, QDs technology is a development after the tracer marker colloidal gold Immunological testing methods (Geszke-Moritz and Moritz, 2013). Fluorescent microspheres are used in different technologies, such as electrochemical technology, immunochromatography technology, and other detection technologies (Shivalkar et al., 2022). Xiang et al. (2011) used self-assembly technology to alternately assemble quantum dot fluorescent microspheres, which were combined with electrochemical methods for the determination of urethral pathogens. They obtained a sensitivity more than ten times higher than other fluorescent substances such as colloidal gold.

In this study, quantum dots were used as labeling materials to label 25-OH VD, and QDs-FICA provided a new detection method for 25-OH VD. We used several methods to verify whether the coupling was successful. High-resolution TEM images of individual particles showed that a large number of oil-soluble ZnCdSe/ZnS QDs were tightly embedded in the polymer. The data indicate that there is effective coupling between the QDs and the 25-OH VD monoclonal antibody, and confirm successful synthesis of the immunofluorescence probe. When mAb are coupled to QDs, the excitation and emission wavelengths remained unchanged, but the fluorescence intensity was weaker than that of QDs, which may be related to the effect of the antibody on the fluorescence detection or the occurrence of fluorescence burst during the coupling process. Although EDC reactions are usually carried out in acidic buffer solutions (pH 4.7–5.5), effective coupling can also be achieved in high-pH buffer solutions. After repeated verification, the optimal pH of ZnCdSe/ZnS core-shell QDs and anti-25-OH VD mAb was 7.6. The ideal reading time of the test strip was 15 min The concentration of 0.2 mol/L of NaC1 solution was determined as the optimal salt ion concentration. A solution with 10% ethanol mass fraction was selected for this experiment. The QDs-FICA exhibited dynamic linear detection of 25-OH VD in PBS from 5 ng/mL to 100 ng/mL and the strip quantitative curves could be represented by the following regression equation: y = −0.02088 logx)+1.444 (*R* ^2^ = 0.9050). The IC_50_ of the QDs-FICA was achieved at 39.6 ± 1.33 ng/mL. The cross-reaction rate between the test strip and 25-OH VD_2_ was 100%, indicating that the affinity of the antibody to 25-OH VD_2_ was equal to 25-OH VD. The cross-reaction rates of 25-OH VD_3,_ 125-OH_2_VD_3_, and125-OH_2_VD_2_ with the strip were 28%, 10%, and 22%, respectively. The cross-reaction rate VD_3_ and VD_2_ were less than 1% with the test strip. The intra-batch spiking recoveries ranged between 85.6% and 90.76%, with coefficients of variance ranging between 7.0% and 23.72%. The recovery rates of the intra- and inter-assays for the spiked samples ranged from 80.48% to 93.41%, and the coefficients of variation were all below 10%. The test strips were used to determine the presence of 25-OH VD in human serum.

These findings indicate that the QDs-FICA exhibit superior performance to colloidal gold; this may be due to the low capture rate of positive results observed by colloidal gold immunochromatography through observation with the naked eye in contrast, fluorescent materials such as QDs can be collected and read by instruments through fluorescence emission, resulting in higher reliability of the results. In addition, the superior performance of QDs enables their widespread application in the field of immunochromatography. The QDs-FICA established in this study is suitable for the rapid clinical detection of vitamin D at the community level and in remote areas. The study also meets the demands for visualization inspection, providing a good choice for screening and surveillance of in the future.

## Conclusion

In summary, in this study, we applied QDs-FICA to 25-OH VD detection for the first time. Compared with traditional immune detection methods, this method provides a new detection method for 25- OH VD. The 25-OH VD magnetic particle chemiluminescence immunoassay established in this study can be applied to quantitative clinical detection of 25-OH VD content in human serum, and its accuracy, precision, linearity, cross-reactivity, anti-interference ability, and stability meet clinical requirements. Besides, the QDs-FICA established in this study is suitable for the rapid clinical detection of vitamin D at the community level and in remote areas, providing a good choice for disease screening and surveillance.

## Data Availability

The original contributions presented in the study are included in the article/Supplementary Material, further inquiries can be directed to the corresponding authors.
