# Supplementary material for: Development of ZnCdSe/ZnS quantum dot-based fluorescence immunochromatographic assay for the rapid visual and quantitative detection 25⁃hydroxyvitamins D in human serum
Source: Front Bioeng Biotechnol. 2023 Dec 22;11:1326254. doi: 10.3389/fbioe.2023.1326254 (PMC10766695; doi:10.3389/fbioe.2023.1326254)
Supplement: Supplementary file 1 [file DataSheet1.docx]

**Supplementary Materials:**


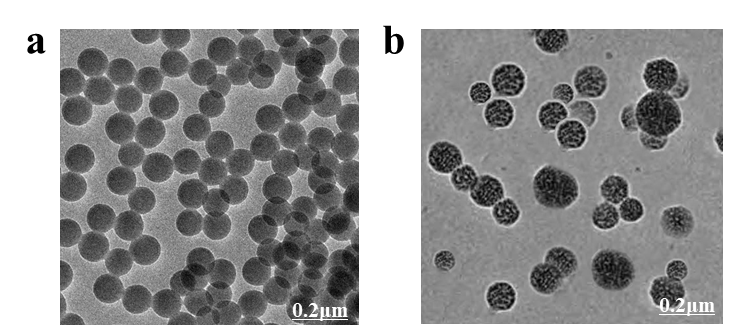


**Supplemental Figure S1 | Transmission electron microscopy of QDs and** **anti-25-OH-VD mAbs- QDs.** (a) Transmission electron microscopy of QDs. (b) Transmission electron microscopy of anti-25-OH-VD mAbs- QDs.

**
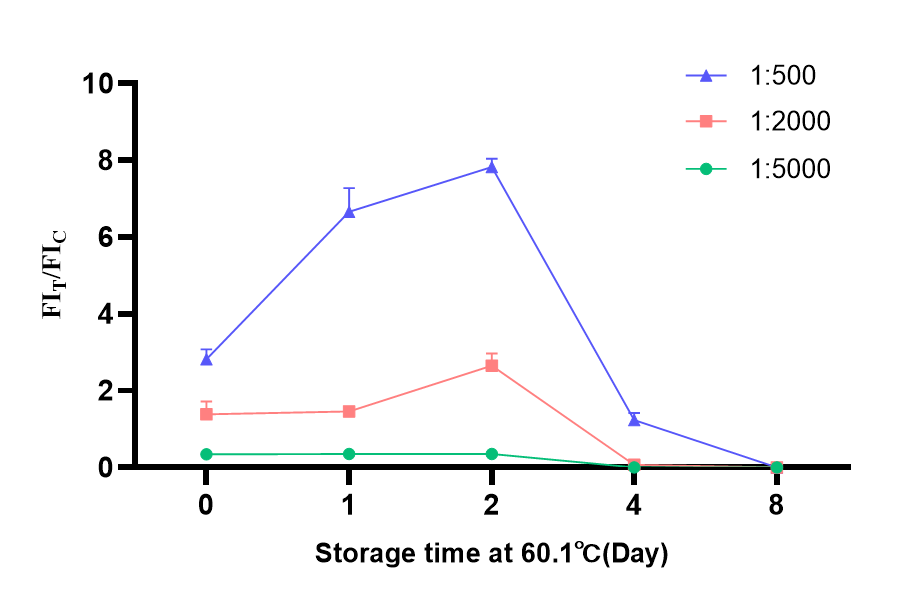
**

**Supplemental Figure S2 | Data graph of stability experiment test.**


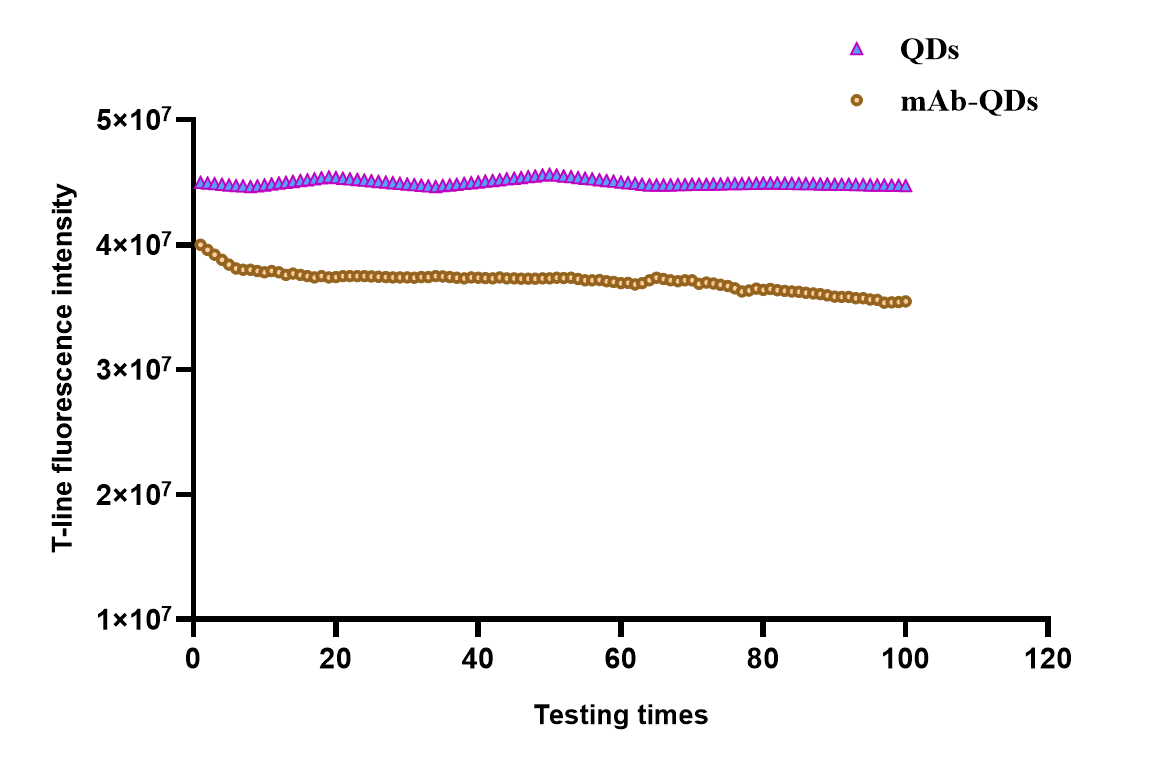
**Supplemental Figure S2 | Fluorescence Stability Test：Continuous Excitation.** The concentration of the film was 0.5mg/mL, and the T-value was measured continuously for 100 times.

**Supplemental Table S1.** Interference species results of 25-OH-VD (n= 3)

| Interference species | Concentration of the added interferents | Mean concentration of interfering samples(ng/mL) | Mean concentration of base sample (ng/mL) | Interference rate (%) |
| --- | --- | --- | --- | --- |
| Biotin | 250 nmol/L | 3.25 | 3.38 | 4.00 |
|  |  | 46.08 | 49.16 | 6.68 |
|  |  | 92.41 | 97.05 | 5.02 |
| Triacylglycerol | 1500 mg/dL | 3.18 | 3.35 | 5.35 |
|  |  | 48.22 | 50.28 | 4.27 |
|  |  | 89.08 | 92.81 | 4.19 |
| Hemoglobin | 1500 mg/dL | 3.3 | 3.51 | 6.36 |
|  |  | 45.79 | 47.87 | 4.54 |
|  |  | 93.23 | 97.13 | 4.18 |
| Bilirubin | 200 mg/dL | 3.19 | 3.40 | 6.58 |
|  |  | 47.53 | 50.09 | 5.39 |
|  |  | 91.67 | 95.57 | 4.25 |
